# Endovascular Therapy vs Medical Management for Patients With Acute Stroke With Medium Vessel Occlusion in the Anterior Circulation

**DOI:** 10.1001/jamanetworkopen.2022.38154

**Published:** 2022-10-24

**Authors:** Hamidreza Saber, Shashvat M. Desai, Diogo Haussen, Alhamza Al-bayati, Shahram Majidi, J. Mocco, Ameer E. Hassan, Gary Rajah, Muhammad Waqas, Jason M. Davies, David Dornbos, Christopher Nickele, Adam S. Arthur, Ashkan Mowla, Matthew S. Tenser, Maxim Mokin, Elliot Pressman, Amin Aghaebrahim, Ricardo A. Hanel, Santiago Ortega-Gutierrez, Tudor Jovin, Gary R. Duckwiler, David S. Liebeskind, Raul G. Nogueira, Jeffrey Gornbein, Jeffrey L. Saver, Ashutosh P. Jadhav

**Affiliations:** 1 Department of Radiology, University of California, Los Angeles; 2 Department of Neuroscience, HonorHealth, Scottsdale, Arizona; 3 Marcus Stroke and Neuroscience Center, Grady Memorial Hospital, Atlanta, Georgia; 4 Department of Neurology, UPMC Stroke Institute, University of Pittsburgh, Pittsburgh, Pennsylvania; 5 Department of Neurosurgery, Icahn School of Medicine at Mount Sinai, New York, New York; 6 Department of Neurology, University of Texas Rio Grande Valley, Harlingen; 7 Department of Neurosurgery, Munson Medical Center, Traverse City, Michigan; 8 Department of Neurosurgery, University at Buffalo, Buffalo, New York; 9 Department of Neurological Surgery, Semmes-Murphey Clinic, University of Tennessee Health Science Center, Memphis; 10 Department of Neurosurgery, University of South California, Los Angeles; 11 Department of Neurosurgery, University of South Florida, Tampa; 12 Baptist Neurological Institute, Lyerly Neurosurgery, Baptist Health, Jacksonville, Florida; 13 Department of Neurology, University of Iowa, Iowa City; 14 Department of Neurology, Cooper University Health Care, Camden, New Jersey; 15 Stroke Center and Department of Neurology, University of California Los Angeles, Los Angeles; 16 Department of Medicine and Computational Medicine, University of California Los Angeles; 17 Barrow Neurological Institute, Phoenix, Arizona

## Abstract

**Importance:**

Randomized clinical trials have shown the efficacy of endovascular therapy (EVT) for acute large vessel occlusion strokes. The benefit of EVT in acute stroke with distal, medium vessel occlusion (DMVO) remains unclear.

**Objective:**

To examine the efficacy and safety outcomes associated with EVT in patients with primary DMVO stroke when compared with a control cohort treated with medical management (MM) alone.

**Design, Setting, and Participants:**

This multicenter, retrospective cohort study pooled data from patients who had an acute stroke and a primary anterior circulation emergency DMVO, defined as any segment of the anterior cerebral artery (ACA) or distal middle cerebral artery, between January 1, 2015, and December 31, 2019. Those with a concomitant proximal occlusion were excluded. Outcomes were compared between the 2 treatment groups using propensity score methods. Data analysis was performed from March to June 2021.

**Exposures:**

Patients were divided into EVT and MM groups.

**Main Outcomes and Measures:**

Main efficacy outcomes included 3-month functional independence (modified Rankin Scale [mRS] scores, 0-2) and 3-month excellent outcome (mRS scores, 0-1). Safety outcomes included 3-month mortality and symptomatic intracranial hemorrhage.

**Results:**

A total of 286 patients with DMVO were evaluated, including 156 treated with EVT (mean [SD] age, 66.7 [13.7] years; 90 men [57.6%]; median National Institute of Health Stroke Scale [NIHSS] score, 13.5 [IQR, 8.5-18.5]; intravenous tissue plasminogen activator [IV tPA] use, 75 [49.7%]; ACA involvement, 49 [31.4%]) and 130 treated with medical management (mean [SD] age, 69.8 [14.9] years; 62 men [47.7%]; median NIHSS score, 7.0 [IQR, 4.0-14.0], IV tPA use, 58 [44.6%]; ACA involvement, 31 [24.0%]). There was no difference in the unadjusted rate of 3-month functional independence in the EVT vs MM groups (151 [51.7%] vs 124 [50.0%]; *P* = .78), excellent outcome (151 [38.4%] vs 123 [31.7%]; *P* = .25), or mortality (139 [18.7%] vs 106 [11.3%]; *P* = .15). The rate of symptomatic intracranial hemorrhage was similar in the EVT vs MM groups (weighted: 4.0% vs 3.1%; *P* = .90). In inverse probability of treatment weighting propensity analyses, there was no significant difference between groups for functional independence (adjusted odds ratio [aOR], 1.36; 95% CI, 0.84-2.19; *P* = .20) or mortality (aOR, 1.24; 95% CI, 0.63-2.43; *P* = .53), whereas the EVT group had higher odds of an excellent outcome (mRS scores, 0-1) at 3 months (aOR, 1.71; 95% CI, 1.02-2.87; *P* = .04).

**Conclusions and Relevance:**

The findings of this multicenter cohort study suggest that EVT may be considered for selected patients with ACA or distal middle cerebral artery strokes. Further larger randomized investigation regarding the risk-benefit ratio for DMVO treatment is indicated.

## Key Points

**Question:**

In patients with acute ischemic stroke and primary distal, medium vessel occlusion (DMVO) in anterior circulation, is endovascular therapy (EVT) associated with improved outcomes at 90 days when compared with patients treated with medical therapy alone?

**Findings:**

In this multicenter cohort study of 286 patients with acute stroke and primary DMVO treated with EVT vs medical therapy alone, no significant difference was found in 90-day functional independence (modified Rankin Scale scores, 0-2), whereas EVT was associated with slightly improved 90-day excellent outcome (modified Rankin Scale scores, 0-1).

**Meaning:**

These findings suggest that EVT may be beneficial in selected patients with primary DMVO.

## Teaser

This cohort study compares the safety and efficacy outcomes associated with endovascular therapy vs medical management alone in the treatment of distal, medium vessel occlusion.

## Introduction

Endovascular therapy (EVT) for cerebral reperfusion has been established as the standard of care for acute large-vessel occlusion (LVO) of the proximal anterior circulation, including the internal carotid artery and the M1 segment of the middle cerebral artery (MCA).^1,2,3,4,5^ Meta-analyses^6,7^ of multicenter prospective cohorts and randomized clinical trials have also suggested benefit of EVT for treatment of occlusions in dominant or codominant branches of the M2 segment of the MCA. However, the safety and efficacy of EVT in primary distal, medium vessel occlusions (DMVOs), such as the M3 segments of the MCA and the anterior cerebral artery (ACA), have not been well delineated because until recently these patients were not enrolled in randomized clinical trials or treated in large volumes in clinical practice. Given the large magnitude of effect and overwhelming benefit of EVT in LVO, mechanical thrombectomy has been postulated to be beneficial in the treatment of DMVOs. However, the smaller size of distal cerebral arteries, as well as longer distances associated with a more tortuous pathway, may increase the difficulty of successful reperfusion in DMVOs.

Patient outcomes with medical management (MM) for DMVOs are similarly not well understood. Between 17% and 40% of patients with acute ischemic stroke (AIS) harbor DMVOs, and a significant proportion of these patients may have poor outcomes.^8,9,10^ Prior single-center cohort studies^11,12,13,14^ have evaluated the role of EVT in patients with DMVOs and suggested a potential benefit with high recanalization and relatively low rates of intracranial hemorrhage after EVT for occlusions in the M3 segment or ACA. However, results of these studies are inconclusive because of small sample sizes, inclusion of patients with initial LVO complicated by emboli to distal or new territories, and lack of a control group with MM alone. To address this gap in knowledge and owing to the lack of randomized clinical trial data, we conducted a multicenter cohort study of registry data to evaluate the safety and efficacy outcomes associated with EVT in primary DMVO strokes when compared with a control cohort treated with medical therapy alone.

## Methods

This multicenter, retrospective cohort study was approved by the relevant ethics committees of individual centers. Informed consent was not required because data were deidentified. This study followed the Strengthening the Reporting of Observational Studies in Epidemiology (STROBE) reporting guideline.^15^

### Study Population

Patients with AIS with DMVO in the M3 segment of the MCA or in the ACA were identified in prospectively maintained data registries from 11 US stroke centers between January 1, 2015, and December 31, 2019. The data were pooled to analyze the safety and efficacy outcomes in patients with DMVO treated with EVT vs MM alone. Patients 18 years or older were included in the analysis with the following criteria: (1) patients with AIS and causative anterior circulation DMVO (M3 segment of the MCA or any segment of the ACS) diagnosed and treated within 24 hours from the last time the patient was known to be doing well before onset of stroke identified, (2) no proximal LVO at presentation, and (3) prestroke modified Rankin Scale (mRS) score of 0 to 1. The mRS assesses disability in patients with stroke and is used as a standard estimate for recovery or degree of continued disability after stroke. A score of 0 indicates no residual symptoms, 1 indicates no significant disability, 5 is severe disability requiring constant care for all needs, and 6 indicates death. The M3 segment was defined from the circular sulcus of the insula to the superior surface of the Sylvian fissure. The patients with DMVO treated with MM alone were gathered from registries from 7 institutions (eTable 1 in the Supplement).

Stroke management decisions, including the delivery of intravenous thrombolytic drugs and hemodynamic management, were made by the patients’ attending physicians and clinical care team after then-current American Heart Association guidelines.^16^ The DMVO strokes were characterized based on initial computed tomography (CT) angiography or magnetic resonance (MR) angiography at presentation, and perfusion images (MR perfusion or CT perfusion) were obtained as part of the standard imaging protocol for all patients along with MR angiography and CT angiography.

Baseline characteristics included age, sex, presenting National Institute of Health Stroke Scale (NIHSS) score, hypertension, diabetes, atrial fibrillation, hyperlipidemia, smoking status, time from when the patient was last known to be doing well to transfer to the emergency department, location of occlusion, and use of intravenous tissue plasminogen activator (tPA). Data on race were not collected because of significant missing values in some centers. ASPECTS (Alberta Stroke Programme Early CT Score) was established as a 10-point topographic score to quantify early ischemic changes in the MCA territory.^17^ Reasons for withholding intravenous tPA included the last time the patient was known to be doing well to the time of presentation greater than 4.5 hours, active use of anticoagulants, history of cerebral hemorrhage, recent intracranial or intraspinal surgery, or active or recent internal bleeding.

We evaluated the following efficacy outcomes: 3-month functional independence defined as mRS scores of 0 to 2 at 90 days, 3-month excellent outcomes defined as mRS scores of 0 to 1 at 90 days, and successful reperfusion. The degree of reperfusion for the medium vessel occlusion vascular territory was determined by the modified Treatment in Cerebral Ischemia (mTICI) score. Successful reperfusion was defined as an mTICI score of 2b (reperfusion of more than half of the previously occluded target artery ischemic territory) or 3 (complete reperfusion).^18^ The safety end points included 3-month all-cause mortality; symptomatic intracranial hemorrhage (sICH) defined as presence of a parenchymal hematoma on follow-up CT with an increase in NIHSS score of at least 4 points within 24 hours from treatment.^19^ A separate analysis was performed among only patients with baseline NIHSS scores greater than 5.

### Statistical Analysis

Analyses were conducted between March and June 2021. Categorical variables were expressed as numbers (percentages) and continuous variables as means (SDs) or medians (IQRs). The *P* values for comparing differences between groups were computed using the Fisher exact test for categorical data and the 2-tailed *t* test and Wilcoxon rank sum test for comparing continuous variables as appropriate.

Because this is not a randomized clinical trial, there was imbalance between the 2 groups on some covariates and potentially confounding factors. Therefore, a prespecified inverse probability of treatment weighting (IPTW) propensity score method was used to correct for imbalance on these prespecified confounding factors when estimating the mean treatment effect size. The weights were computed using a logistic regression model in which treatment was the outcome and age, sex, presenting NIHSS score, atrial fibrillation, diabetes, intravenous tPA, and the last time the patient was known to be doing well before transfer to the emergency department were the covariates. The association of treatment (EVT vs MM) with outcomes was then evaluated using an IPTW logistic model for the binary outcome where robust SEs were computed for the regression coefficients (log odds ratios [ORs]) by clustering on subclass for which a unique subclass value is assigned to each subgroup (pair) of EVT-MM observations that have the same weight. Unweighted vs weighted balance between the EVT and MM groups was assessed by computing standardized mean differences (mean difference divided by pooled SD) for each covariate and the variance ratio for each covariate without weighting vs these values using the IPTW. A standardized mean difference greater than 0.25 was considered meaningful. Odds ratios and 95% CIs and *P* values based on the IPTW logistic model for EVT vs MM binary outcome are reported. A 2-sided *P* < .05 was considered statistically significant.

Eleven individuals with missing functional outcomes and 3 with missing sICH outcomes were excluded from the propensity score analyses. Missing data for independent variables were identified as greater than 1% for the last time the patient was known to be doing well before transfer to the emergency department (missing completely at random). The values for this variable were imputed using multiple imputation via chained equations.^20^ All statistical analysis was performed using Stata software, version 13.1 (StataCorp LLC) and R software, version 4.0.5 (R Foundation for Statistical Computing).

## Results

**Figure 1.**
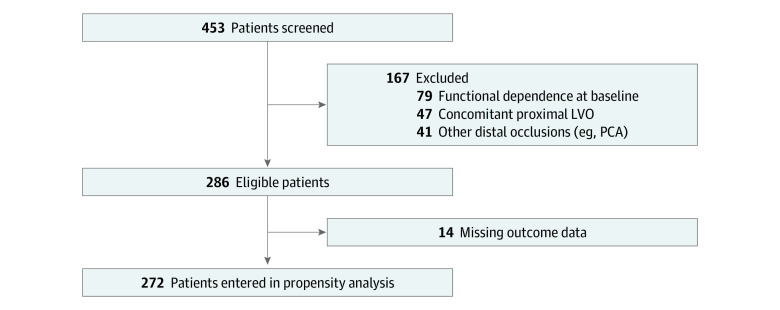
Cohort Build for Acute Stroke With Distal, Medium Vessel Occlusion Treated With Endovascular Therapy vs Medical Management Alone LVO indicates large vessel occlusion; PCA, posterior cerebral artery.

**Figure 2.**
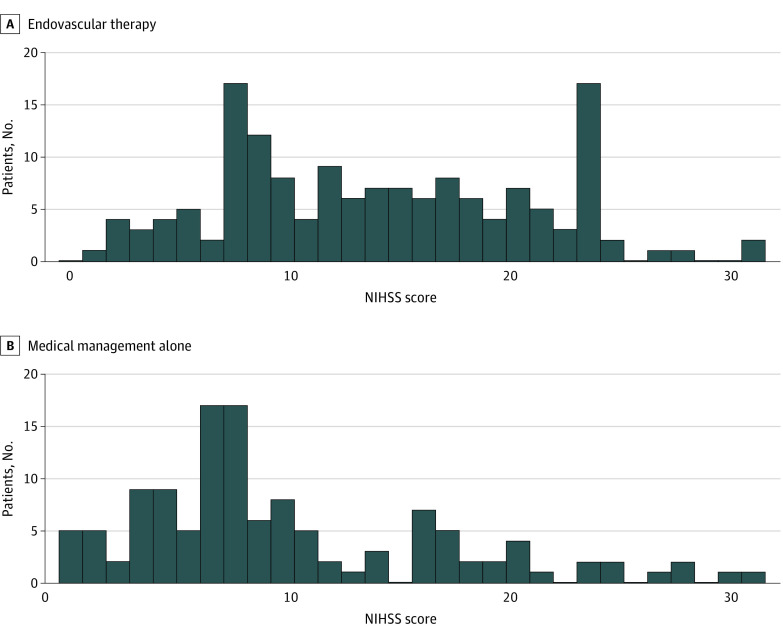
Distribution of National Institute of Health Stroke Scale (NIHSS) Scores Among Included Patients With M3 Segment Middle Cerebral Artery and Anterior Cerebral Artery Occlusion by Treatment Group

A total of 286 patients with DMVO met study eligibility criteria, including 156 treated with EVT (mean [SD] age, 66.7 [13.7] years; 90 men [57.6%] and 66 women [42.3%]) and 130 with MM alone (mean [SD] age, 69.8 [14.9] years; 62 men [47.7%] and 68 women [52.3%]). The median (IQR) baseline NIHSS score was significantly higher in the EVT group compared with the MM group (13.5 [8.5-18.5] vs 7 [4-14]; *P* < .001). The rate of intravenous tPA administration did not significantly differ between the 2 groups (75 [49.7%] in the EVT group and 58 [44.6%] in the MM group; *P* = .39) (Table 1). Among 80 patients presenting with ACA DMVO (49 [31.4%] in the EVT group and 31 [24.0%] in the MM group), 29 (36.2%) had A1 occlusion and 51 (63.8%) had A2 to A3 occlusions. Figure 1 shows the flow diagram of the study and the propensity score analysis. Figure 2 shows the overall NIHSS score distribution of patients with M3 or ACA occlusion. Overall, successful recanalization was achieved in 118 patients (81.2%) with AIS with DMVO after EVT. Aspiration alone was used in 76 patients (49.0%), stent-retriever EVT (with or without aspiration) was used in 67 (43.5%), and 12 (7.5%) were treated with intra-arterial alteplase. First-pass recanalization with mTICI scores of 2b or 3 was performed in 88 patients (56.6%) after EVT. The rate of sICH was similar in the EVT and MM groups(weighted: 4.0% vs 3.1%; *P* = .54).

**Table 1.** Comparison of Clinical Characteristics and Unadjusted Outcomes in All Patients With M3 MCA or ACA Occlusions Treated With EVT or MM

Variable	EVT group (n = 156)	MM group (n = 130)	*P* value
Age, mean (SD), y	66.7 (13.7)	69.8 (14.9)	.001
Sex, No. (%)			
Male	90 (57.6)	62 (47.7)	.10
Female	66 (42.3)	68 (52.3)	
Baseline NIHSS score			
Mean (SD)	13.9 (6.8)	9.4 (7.2)	<.001
Median (IQR)	13.5 (8.5-18.5)	7.0 (4.0-14.0)	
NIHSS score, weighted %			
<10 (n = 132)	38.6	61.4	<.001
10-19 (n = 101)	65.4	34.6	
>19 (n = 53)	73.6	26.4	
Intravenous tPA use, No. (%)	75 (49.7)	58 (44.6)	.39
Medical history and risk factor (presence), No. (%)			
Hypertension	132 (84.6)	101 (77.8)	.13
Atrial fibrillation	60 (38.5)	34 (26.2)	.03
Diabetes	44 (28.6)	35 (27.1)	.75
Dyslipidemia	79 (57.4)	65 (54.8)	.72
Occlusion site			
MCA (M3 segment)	107 (68.5)	101 (77.7)	.11
ACA	49 (31.4)	31 (24.0)	.16
Baseline ASPECTS, median (IQR)	9 (7-10)	9 (8-10)	.56
Time from when the patient was last known to be well to transfer to the emergency department, mean (SD), min	266.8 (207.61)	241.9 (202.9)	.40
General anesthesia, No. (%)	22 (15.1)	NA	NA
Revascularization (mTICI 2b+), No. (%)	118 (81.2)	NA	NA

Abbreviations: ACA, anterior cerebral artery; ASPECTS, Alberta Stroke Program Early CT Score; EVT, endovascular therapy; MCA, middle cerebral artery; MM, medical management; mTICI, modified Treatment in Cerebral Ischemia; NA, not applicable; tPA, tissue plasminogen activator.

The distribution of 90-day mRS scores by treatment group is demonstrated in eFigure 1 in the Supplement. In unadjusted analysis of the full cohort, rates were not significantly different between the 2 groups for 3-month functional independence (151 [51.7%] with EVT vs 124 [50.0%] with MM; *P* = .78) and excellent outcome (151 [38.4%] vs 123 [31.7%]; *P* = .25).

The rates of sICH did not significantly differ between the EVT vs MM groups in the full cohort in unadjusted analysis (156 [3.8%] vs 127 [3.1%]; *P* = .90). Similarly, no significant differences were observed in 3-month mortality outcomes between the EVT and MM groups in unadjusted analysis (139 [18.7%] vs 106 [11.3%]; *P* = .15) (Table 2).

**Table 2.** Comparisons of Clinical Outcomes After EVT vs MM of Medium Vessel Occlusion Before and After Inverse Probability Weighting Adjustment

Outcome	MM group		EVT group		Risk difference, mean (SE), %		OR (95% CI)	*P* value
	No. (%)	Weighted, %	No. (%)	Weighted,%				
**Functional independence**								
Unadjusted	124 (50.0)	4.5	151 (51.7)	4.1	1.7 (6.1)		1.07 (0.66-1.72)	.78
Adjusted	124 (44.0)	4.5	151 (51.7)	4.1	7.7 (6.0)		1.36 (0.84-2.19)	.20
**Excellent outcome**								
Unadjusted	123 (31.7)	4.2	151 (38.4)	4.0	6.7 (5.8)		1.34 (0.79-2.29)	.25
Adjusted	123 (26.7)	4.0	151 (38.4)	4.0	11.7 (5.6)		1.71 (1.02-2.87)	.04
**Mortality**								
Unadjusted	106 (11.3)	3.1	139 (18.7)	3.3	7.4 (4.5)		1.80 (0.82-4.14)	.15
Adjusted	106 (15.7)	3.5	139 (18.7)	3.3	3.0 (4.8)		1.24 (0.63-2.43)	.53
**sICH** ^a^								
Unadjusted	127 (3.1)	1.5	156 (3.8)	1.5	0.7 (2.2)		1.23 (0.28-6.06)	.90

Abbreviations: EVT, endovascular therapy; MM, medical management; OR, odds ratio; sICH, symptomatic intracranial hemorrhage.

^a^ Adjusted outcomes were not computed for sICH because of low number of outcome events.

### Propensity Score Analysis Using IPTW

The propensity score method among patients yielded EVT and MM groups highly similar in baseline characteristics. eTable 2 and eTable 3 in the Supplement show balance between the EVT vs MM groups by demonstrating standardized mean differences for each covariate and the variance ratio before and after IPTW. The Love plot is shown in eFigure 2 in the Supplement. In IPTW-adjusted analyses, no significant difference was found between treatment groups for the primary outcome of functional independence (adjusted OR [aOR], 1.36; 95% CI, 0.84-2.19; *P* = .20), whereas EVT was associated with a higher odds of an excellent outcome at 3 months (aOR, 1.71; 95% CI, 1.02-2.87; *P* = .04) (Table 2). In IPTW analysis, no significant difference was observed for 3-month mortality between treatment groups (aOR), 1.24; 95% CI, 0.63-2.43; *P* = .53). Adjusted analyses were not performed for the sICH outcome because of the low number of events. The propensity-weighted proportions for the primary outcome of functional independence were 44.0% in the MM group and 51.7% in the EVT group.

In the IPTW analysis restricted to those with baseline NIHSS scores greater than 5, no significant difference was found between treatment groups for the outcome of functional independence (aOR, 1.27; 95% CI, 0.74-2.19; *P* = .38), whereas EVT was associated with a higher likelihood of excellent outcomes (aOR, 1.86; 95% CI, 1.00-3.44; *P* = .05).

### EVT for AIS With ACA vs M3 MCA Occlusions

The age and number of men were similar among patients with ACA (mean [SD] age, 69.8 [10.3] years; 28 [57.1%] male) vs M3 MCA (mean [SD] age, 65.5 [14.7] years; 62 [57.9%] male) occlusions who underwent EVT. The presenting NIHSS score was comparable between AIS patients with ACA (mean [SD], 13.0 [6.3]) vs M3 MCA (mean [SD], 14.2 [7.1]) occlusions undergoing EVT (Table 3). Successful recanalization was achieved in 86.1% after ACA and 79.3% after M3 MCA EVT (*P* = .12). First-pass successful reperfusion (mTICI 2b-3) was achieved in 60.1% of patients with AIS with M3 occlusions and 53.2% of AIS with ACA occlusions (*P* = .41). The total pass numbers were higher in ACA vs M3 MCA EVT (mean [SD], 2.1 [1.8] vs 1.5 [1.3] ; *P* = .03). The rate of sICH was similar for ACA vs M3 MCA EVT (2 [4.1%] vs 4 [3.7%]; *P* = .62); however, the rate of 3-month functional independence tended to be lower in patients with ACA compared with those with M3 MCA EVT, but the difference was not statistically significant (19 [40.4%] vs 59 [56.7%]; *P* = .06) (Table 3). Similarly, among patients in the MM group, the rate of 3-month functional independence (weighted: 35.4% vs 55.0%; *P* = .06) and excellent outcomes (29.0% vs 34.1%; *P* = .55) were proportionally lower but not significantly different in patients with ACA compared with those with M3 MCA strokes.

**Table 3.** Comparison of Baseline Clinical Characteristics and Unadjusted Outcomes in Patients With M3 MCA vs ACA Occlusions Undergoing Endovascular Therapy

Characteristic	ACA (n = 49)	MCA (n = 107)	*P* value
Age, mean (SD), y	69.8 (10.3)	65.5 (14.7)	.10
Sex, No. (%)			
Male	28 (57.1)	62 (57.9)	.90
Female	21 (42.9)	45 (42.1)	
Baseline NIHSS score, mean (SD)	13.0 (6.3)	14.2 (7.1)	.30
Baseline NIHSS score, weighted %			
<10	44.6	55.5	<.001
10-20	68.7	31.3	
>20	55.8	44.2	
Intravenous tPA, No. (%)	15 (31.9)	60 (57.7)	.01
No. of passes, mean (SD)	2.1 (1.8)	1.5 (1.3)	.03
Medical history and risk factors, weighted %			
Hypertension	87.7	79.4	.04
Atrial fibrillation	34.7	40.2	.38
Diabetes	38.3	24.3	.04
Dyslipidemia	67.5	44.4	.03
First-pass recanalization	53.6	60.2	.45
General anesthesia	9.5	16.0	.25
Postprocedural outcomes			
Revascularization (mTICI 2b+), weighted %	86.1	79.3	.12
sICH, No. (%)	2 (4.1)	4 (3.7)	.62
90-Day outcome, No. (%)			
Good (mRS scores, 0-2)	19 (40.4)	59 (56.7)	.06
Excellent (mRS scores, 0-1)	15 (31.9)	43 (41.3)	.40

Abbreviations: ACA, anterior cerebral artery; MCA, middle cerebral artery; mRS, modified Rankin Scale; mTICI, modified Treatment in Cerebral Ischemia; NIHSS, National Institute of Health Stroke Scale; sICH, symptomatic intracerebral hemorrhage; tPA, tissue plasminogen activator.

## Discussion

This multicenter cohort study supports the feasibility and safety of EVT in patients with AIS with DMVO. In our study, patients with DMVO who underwent EVT were more likely to be younger and present with a higher NIHSS score at onset compared with those treated with MM alone. Successful reperfusion was achieved in more than 80% of the patients with DMVO. After propensity score adjustment for basic characteristics, including age and presenting NIHSS score, no significant difference was found in the rate of 3-month functional independence between the treatment groups; however, EVT was associated with a greater likelihood of achieving an excellent outcome compared with MM alone. In terms of safety, no significant differences were found in the risk of mortality or sICH with EVT compared with MM alone across patients with DMVO.

Given the extensive randomized clinical trial evidence base, current American Stroke Association guidelines regarding EVT focus heavily on large vessel occlusion strokes. The 2019 American Stroke Association guidelines indicate it “may be reasonable” (class IIb recommendation) to perform EVT for MCA M3 occlusions and offer no EVT-related recommendations for MCA M4 and ACA occlusions.^15^ European guidelines do not provide specific recommendations regarding M3, M4, and ACA occlusions.^21^ To our knowledge, this study is the first multicenter study describing and comparing outcomes in patients with AIS receiving EVT vs MM alone for primary occlusions of the M3 MCA and ACA distal and medium vessels in the anterior circulation. Strokes with DMVO were associated with high rates of disability regardless of treatment, with dependency or worse outcome in 5 of every 10 patients; mortality by 3 months accrued in nearly 2 of every 10 EVT-treated patients and 1 of every 10 medically treated patients.

Although the likelihood of recanalization with intravenous thrombolysis is increased in more distal cerebral vessels with smaller calibers, approximately half of patients with DMVO do not achieve early reperfusion after intravenous tPA administration.^22^ The current study shows that a high rate of successful reperfusion (>8 of every 10 patients) can be achieved in DMVO strokes with EVT with no significant increase in sICH. Furthermore, as shown previously and supported in the current analysis, DMVO strokes are not benign and represent an opportunity to maximize the impact of EVT to further improve outcomes.^9^ Randomized clinical trials will be needed to confirm these findings and to identify patients with DMVO who are more likely to benefit from EVT.

Further indicating a potential benefit of EVT in patients with DMVO stroke, the rates of successful reperfusion, sICH, and mortality in the current study were similar to those in a pooled analysis of the pivotal trials of EVT for LVO strokes, and the rates of 3-month functional independence were higher.^23^ These findings suggest that with modern endovascular technology, EVT procedures in more distal and fragile DMVO can be performed as safely as for LVOs. Our results are not generalizable to strokes associated with DMVOs other than ACA or M3 MCA territories.

Until recently, distal or medium vessel occlusions were not a frequent target of endovascular intervention. Less frequent use of vessel imaging and a lack of appropriate endovascular devices made EVT for primary DMVO uncommon. In the IMS-III trial (Interventional Management of Stroke III), only 5 patients with M3, M4, or ACA occlusions were included.^24^ In the MR CLEAN trial (Multicenter Randomized Clinical Trial of Endovascular Treatment for Acute Ischemic Stroke in the Netherlands), only 3 ACA occlusions were included.^2^ However, with technologic advances, experience with EVT for DMVO stroke is beginning to expand.^9,10^ Grossberg et al^11^ studied distal intracranial occlusion strokes, including ACA, M3 MCA, and posterior cerebral artery occlusions, and reported that EVT was effective in achieving successful reperfusion in 83% of cases, with a 3-month functional independence (mRS scores, 0-2) rate of 30% and a 3-month mortality rate of 20%. The current study of a larger, multicenter population shows similar reperfusion and mortality rates and higher rates of functional independence. This study additionally offers dual insights into the outcome of DMVO strokes under MM alone as well as with EVT and can inform the design of future trials investigating the optimal endovascular vs medical treatment of DMVO.

Several additional practical considerations need to be revisited in the context of EVT for DMVO strokes. Current prehospital stroke severity triage scales were developed and calibrated to identify LVOs rather than DMVOs.^25,26^ Current interhospital transfer paradigms similarly were developed to identify and rapidly transfer patients with LVO rather than patients with DMVO ischemic stroke. Future trials could assess the relevance of perfusion-based neuroimaging paradigms, including physician (level of skill to detect distal occlusions on vessel imaging) and technology-related factors (added utility of perfusion imaging or time variant CT angiography) for selection and treatment of DMVO strokes. Newer devices with smaller diameters and very-low-profile thrombectomy devices are being developed for DMVOs.^14,27^ The role of anesthesia in DMVO EVT will need to be studied because general anesthesia may be preferred in DMVO to increase the safety profile of EVT in these distal, smaller, and more fragile vessels. The association of tenecteplase with treatment of DMVO strokes will need to be thoroughly investigated. The wide range of stroke severity at onset in patients with M3 MCA occlusions could represent differences in eloquence of each of these branches. Patients with DMVO in the central or precentral branches (supplying the primary motor cortex) may present with higher stroke severity compared with patients with DMVO involving less eloquent regions, such as parietal or angular branches. Patient selection for EVT based on the eloquence of the occluded branch in addition to stroke severity at onset should also be considered in future studies. Currently, there is no standard approach or guideline across different institutions and stroke centers for treatment of DMVO. In our study, the main driving factors for differences in treatment strategy were likely related to institutional and interventionalist variation in patient selection across different centers. Routinely used ASPECTS, mTICI, or mRS scores may not be adequate for outcome adjudication after DMVO strokes. The modest effect size associated with EVT in the adjusted analysis may also be related to the milder presentations in DMVO and smaller differences among treatment groups. In line with our findings for anterior circulation DMVO, a recent study^28^ suggested that EVT for posterior circulation DMVO is safe and technically feasible.

### Limitations

Our study is limited by its nonrandomized nature. Patients treated with EVT presented with a higher baseline deficit and were at a higher risk of developing worse outcomes compared with the MM group. This finding is also reflected in the lower magnitude of the ORs in the crude analyses vs the analysis based on IPTW. The IPTW analyses mitigate this concern, but the possibility of residual or unmeasured confounding remains significant. Therefore, our study may have underestimated the real odds of improved outcome in the EVT vs the MM group, which can be assessed in future randomized trials. The current study was confined to M3 MCA and ACA DMVO and did not investigate DMVO in other arterial segments, including nondominant M2 MCA branches and the posterior cerebral artery. The mTICI and ASPECT scores were adjudicated blinded to clinical outcomes; however, clinical outcomes and ascertainment of hemorrhagic conversion were not assessed blinded to the mode of treatment (EVT vs MM alone). A difference was found in number of sites that contributed to the EVT and MM patient cohorts. Our sample size is small when compared with the landmark trials of the large vessel occlusion strokes, primarily because DMVO strokes represent a smaller number of disabling strokes and are less frequently treated based on current guidelines. With the assumption of a 31.7% rate of an excellent outcome under MM, a sample size of 364 per group would be needed to provide 80% power for confirming a 10% improvement in the excellent outcome from 31.7% to 41.7% using the usual 2-sided *P* < .05 significance criterion; therefore, our study is underpowered to detect an accurate OR of the treatment effect. However, our study represents the largest study of its kind involving cases from multiple high-volume stroke centers. Data from patients receiving MM were not collected from 4 centers, and the difference in unmeasured covariates across these centers could have introduced the risk of selection bias. Furthermore, we have attempted to introduce uniformity in our sample by including only anterior circulation strokes treated up to 24 hours after stroke onset.

## Conclusions

In conclusion, the findings of this multicenter cohort study suggest that EVT is safe and may be associated with higher rates of excellent outcome in patients with ischemic stroke due to M3 MCA and ACA medium vessel occlusions. Further investigation into the risks and benefits of DMVO treatment is warranted.

## Notes

Supplement. **eTable 1.** Identification of the Study Registries and Primary Contribution to the Treatment Arms and Imaging Modalities in Cohort **eTable 2.** Baseline Characteristics According to the Use of EVT vs MMA Before IPTW **eTable 3.** Baseline Characteristics According to the Use of EVT vs MMA After IPTW **eFigure 1.** Distribution of 90-Day mRS Scores by Treatment Group **eFigure 2.** Love Plot

## References

[1] Saver JL, Goyal M, Bonafe A, ; SWIFT PRIME Investigators. Stent-retriever thrombectomy after intravenous t-PA vs. t-PA alone in stroke. N Engl J Med. 2015;372(24):2285-2295. doi:10.1056/NEJMoa1415061 25882376

[2] Berkhemer OA, Fransen PSS, Beumer D, ; MR CLEAN Investigators. A randomized trial of intraarterial treatment for acute ischemic stroke. N Engl J Med. 2015;372(1):11-20. doi:10.1056/NEJMoa1411587 25517348

[3] Jovin TG, Chamorro A, Cobo E, ; REVASCAT Trial Investigators. Thrombectomy within 8 hours after symptom onset in ischemic stroke. N Engl J Med. 2015;372(24):2296-2306. doi:10.1056/NEJMoa1503780 25882510

[4] Goyal M, Demchuk AM, Menon BK, ; ESCAPE Trial Investigators. Randomized assessment of rapid endovascular treatment of ischemic stroke. N Engl J Med. 2015;372(11):1019-1030. doi:10.1056/NEJMoa1414905 25671798

[5] Campbell BCV, Mitchell PJ, Kleinig TJ, ; EXTEND-IA Investigators. Endovascular therapy for ischemic stroke with perfusion-imaging selection. N Engl J Med. 2015;372(11):1009-1018. doi:10.1056/NEJMoa1414792 25671797

[6] Saber H, Narayanan S, Palla M, . Mechanical thrombectomy for acute ischemic stroke with occlusion of the M2 segment of the middle cerebral artery: a meta-analysis. J Neurointerv Surg. 2018;10(7):620-624. doi:10.1136/neurintsurg-2017-013515 29127196

[7] Menon BK, Hill MD, Davalos A, . Efficacy of endovascular thrombectomy in patients with M2 segment middle cerebral artery occlusions: meta-analysis of data from the HERMES Collaboration. J Neurointerv Surg. 2019;11(11):1065-1069. doi:10.1136/neurintsurg-2018-014678 30975736

[8] Desai SM, Starr M, Molyneaux BJ, Rocha M, Jovin TG, Jadhav AP. Acute ischemic stroke with vessel occlusion-prevalence and thrombectomy eligibility at a comprehensive stroke center. J Stroke Cerebrovasc Dis. 2019;28(11):104315. doi:10.1016/j.jstrokecerebrovasdis.2019.104315 31409537

[9] Saver JL, Chapot R, Agid R, ; Distal Thrombectomy Summit Group. Thrombectomy for distal, medium vessel occlusions: a consensus statement on present knowledge and promising directions. Stroke. 2020;51(9):2872-2884. doi:10.1161/STROKEAHA.120.028956 32757757

[10] Ospel JM, Goyal M. A review of endovascular treatment for medium vessel occlusion stroke. J Neurointerv Surg. 2021;13(7):623-630. doi:10.1136/neurintsurg-2021-017321 33637570

[11] Grossberg JA, Rebello LC, Haussen DC, . Beyond large vessel occlusion strokes: distal occlusion thrombectomy. Stroke. 2018;49(7):1662-1668. doi:10.1161/STROKEAHA.118.020567 29915125

[12] Chung GH, Kwak HS, Park JS, Lee JM. Manual aspiration thrombectomy with a Penumbra catheter for acute anterior cerebral artery occlusion. Interv Neuroradiol. 2017;23(4):416-421. doi:10.1177/1591019917702521 28443484 PMC5684899

[13] Pfaff J, Herweh C, Pham M, . Mechanical thrombectomy of distal occlusions in the anterior cerebral artery: recanalization rates, periprocedural complications, and clinical outcome. AJNR Am J Neuroradiol. 2016;37(4):673-678. doi:10.3174/ajnr.A4594 26542233 PMC7960160

[14] Rikhtegar R, Mosimann PJ, Weber R, . Effectiveness of very low profile thrombectomy device in primary distal medium vessel occlusion, as rescue therapy after incomplete proximal recanalization or following iatrogenic thromboembolic events ischemic stroke. J NeuroIntervent Surg. 2021;0:1-6. doi:10.1136/neurintsurg-2020-017035 PMC8606433 33468609

[15] von Elm E, Altman DG, Egger M, Pocock SJ, Gøtzsche PC, Vandenbroucke JP; STROBE Initiative. The Strengthening the Reporting of Observational Studies in Epidemiology (STROBE) statement: guidelines for reporting observational studies. PLoS Med. 2007;4(10):e296.doi:10.1371/journal.pmed.0040296 17941714 PMC2020495

[16] Powers WJ, Rabinstein AA, Ackerson T, . Guidelines for the early management of patients with acute ischemic stroke: 2019 update to the 2018 guidelines for the early management of acute ischemic stroke a guideline for healthcare professionals from the American Heart Association/American Stroke Association. Stroke. 2019;50(12):e344-e418. doi:10.1161/STR.0000000000000211 31662037

[17] Barber PA, Demchuk AM, Zhang J, Buchan AM. Validity and reliability of a quantitative computed tomography score in predicting outcome of hyperacute stroke before thrombolytic therapy. ASPECTS Study Group. Alberta Stroke Programme Early CT Score. Lancet. 2000;355(9216):1670-1674. doi:10.1016/S0140-6736(00)02237-6 10905241

[18] Zaidat OO, Yoo AJ, Khatri P, ; Cerebral Angiographic Revascularization Grading (CARG) Collaborators; STIR Revascularization working group; STIR Thrombolysis in Cerebral Infarction (TICI) Task Force. Recommendations on angiographic revascularization grading standards for acute ischemic stroke: a consensus statement. Stroke. 2013;44(9):2650-2663. doi:10.1161/STROKEAHA.113.001972 23920012 PMC4160883

[19] Hacke W, Kaste M, Bluhmki E, ; ECASS Investigators. Thrombolysis with alteplase 3 to 4.5 hours after acute ischemic stroke. N Engl J Med. 2008;359(13):1317-1329. doi:10.1056/NEJMoa0804656 18815396

[20] Van Buuren S, Groothuis-Oudshoorn K. mice: multivariate imputation by chained equations in R. J Stat Softw. 2011;45(3):1-67. doi:10.18637/jss.v045.i03

[21] Turc G, Bhogal P, Fischer U, . European Stroke Organisation (ESO) - European Society for Minimally Invasive Neurological Therapy (ESMINT) Guidelines on Mechanical Thrombectomy in Acute Ischemic Stroke. J Neurointerv Surg. Published online February 26, 2019. doi:10.1136/neurintsurg-2018-014568 31152058

[22] Seners P, Turc G, Maïer B, Mas JL, Oppenheim C, Baron JC. Incidence and predictors of early recanalization after intravenous thrombolysis: a systematic review and meta-analysis. Stroke. 2016;47(9):2409-2412. doi:10.1161/STROKEAHA.116.014181 27462117

[23] Goyal M, Menon BK, van Zwam WH, ; HERMES collaborators. Endovascular thrombectomy after large-vessel ischaemic stroke: a meta-analysis of individual patient data from five randomised trials. Lancet. 2016;387(10029):1723-1731. doi:10.1016/S0140-6736(16)00163-X 26898852

[24] Broderick JP, Palesch YY, Demchuk AM, ; Interventional Management of Stroke (IMS) III Investigators. Endovascular therapy after intravenous t-PA versus t-PA alone for stroke. N Engl J Med. 2013;368(10):893-903. doi:10.1056/NEJMoa1214300 23390923 PMC3651875

[25] Nguyen TTM, van den Wijngaard IR, Bosch J, . Comparison of prehospital scales for predicting large anterior vessel occlusion in the ambulance setting. JAMA Neurol. 2021;78(2):157-164. doi:10.1001/jamaneurol.2020.4418 33252631 PMC8015863

[26] Noorian AR, Sanossian N, Shkirkova K, ; FAST-MAG Trial Investigators and Coordinators. Los Angeles motor scale to identify large vessel occlusion prehospital validation and comparison with other screens. Stroke. 2018;49(3):565-572. doi:10.1161/STROKEAHA.117.019228 29459391 PMC5829024

[27] Gruber P, Diepers M, von Hessling A, . Mechanical thrombectomy using the new Tigertriever in acute ischemic stroke patients—a Swiss prospective multicenter study. Interv Neuroradiol. 2020;26(5):598-601. doi:10.1177/1591019920946499 32720822 PMC7645173

[28] Meyer L, Stracke CP, Jungi N, . Thrombectomy for primary distal posterior cerebral artery occlusion stroke: the TOPMOST Study. JAMA Neurol. 2021;78(4):434-444. doi:10.1001/jamaneurol.2021.0001 33616642 PMC7900924

