# Supplementary material for: Endovascular Therapy vs Medical Management for Patients With Acute Stroke With Medium Vessel Occlusion in the Anterior Circulation
Source: JAMA Netw Open. 2022 Oct 24;5(10):e2238154. doi: 10.1001/jamanetworkopen.2022.38154 (PMC9593229; doi:10.1001/jamanetworkopen.2022.38154)
Supplement: Supplement. — eTable 1. Identification of the Study Registries and Primary Contribution to the Treatment Arms and Imaging Modalities in Cohort eTable 2. Baseline Characteristics According to the Use of EVT vs MMA Before IPTW eTable 3. Baseline Characteristics According to the Use of EVT vs MMA After IPTW eFigure 1. Distribution of 90-Day mRS Scores by Treatment Group eFigure 2. Love Plot [file jamanetwopen-e2238154-s001.pdf]

Supplementary Online Content

Saber H, Desai SM, Haussen D, et al. Endovascular therapy vs medical management for patients with acute stroke with medium vessel occlusion in the anterior circulation. *JAMA Netw Open*. 2022;5(10):e2238154. doi:10.1001/jamanetworkopen.2022.38154

eTable 1. Identification of the Study Registries and Primary Contribution to the Treatment Arms and Imaging Modalities in Cohort

eTable 2. Baseline Characteristics According to the Use of EVT vs MMA Before IPTW

eTable 3. Baseline Characteristics According to the Use of EVT vs MMA After IPTW

eFigure 1. Distribution of 90-Day mRS Scores by Treatment Group

eFigure 2. Love Plot

This supplementary material has been provided by the authors to give readers additional information about their work.

© 2022 Saber H et al. *JAMA Network Open.*

eTable 1. Identification of the Study Registries and Primary Contribution to the Treatment Arms and Imaging Modalities in Cohort

EVT: Endovascular therapy; CTA: CT angiogram; MRA: MR angiogram

**Site**	EVT cohort	Medical treatment cohort	CTA	MRA
Baptist Jacksonville, FL	4	0	*	
Grady Memorial Hospital	39	16	*	
Mount Sinai Hospital	34	0	*	
UCLA Medical Center	3	60	*	*
University at Buffalo	7	7	*	
University of Iowa	2	0	*	
University of Pittsburgh Medical Center	16	16	*	*
University of South California	5	11	*	
University of South Florida	3	8	*	
University of Texas Rio Grande Valley, TX	33	12	*	
University of Tennessee Health Science Center	10	0	*	

© 2022 Saber H et al. *JAMA Network Open.*

eTable 2. Baseline Characteristics According to the Use of EVT vs MMA Before IPTW

	**MM, n=124**		**EVT, n=151**		**EVT-MM**	
**covariate**	**mean or prop**	**SD**	**mean or prop**	**SD**	**std mean diff**	**variance ratio**
age	72.19	14.87	66.54	13.80	-0.41	0.86
gender	48.4%		57.6%		0.18	
NIHSS	9.73	7.14	13.97	6.79	0.62	0.90
Afib	26.6%		39.7%		0.26	
tPA use	45.2%		50.6%		0.11	
last well known to ED	247.92	221.8	270.07	189.96	0.11	0.73
propensity score	0.458	0.19	0.62	0.15	0.99	0.65

MM: Medical management; EVT: Endovascular therapy; Afib: Atrial fibrillation; SD: standard deviation; Prop: proportion

eTable 3. Baseline Characteristics According to the Use of EVT vs MMA After IPTW

	MM,n=124		EVT., n=151		EVT-MM	
covariate	**mean or prop**	**SD**	**mean or prop**	**SD**	**std mean diff**	**variance ratio**
Age	65.06	14.18	66.54	13.80	0.10	0.89
gender	58.6%		57.6%		-0.02	
NIHSS	13.40	7.24	13.97	6.79	0.08	0.75
Afib	34.5%		39.7%		0.10	
tpa use	38.8%		50.6%		0.23	
last well known to ED	300.74	212.5	270.07	189.96	-0.16	0.59
propensity score	0.62	0.16	0.62	0.16	0.01	1.03

MM: Medical management; EVT: Endovascular therapy; Afib: Atrial fibrillation; SD: standard deviation; Prop: proportion

© 2022 Saber H et al. *JAMA Network Open.*

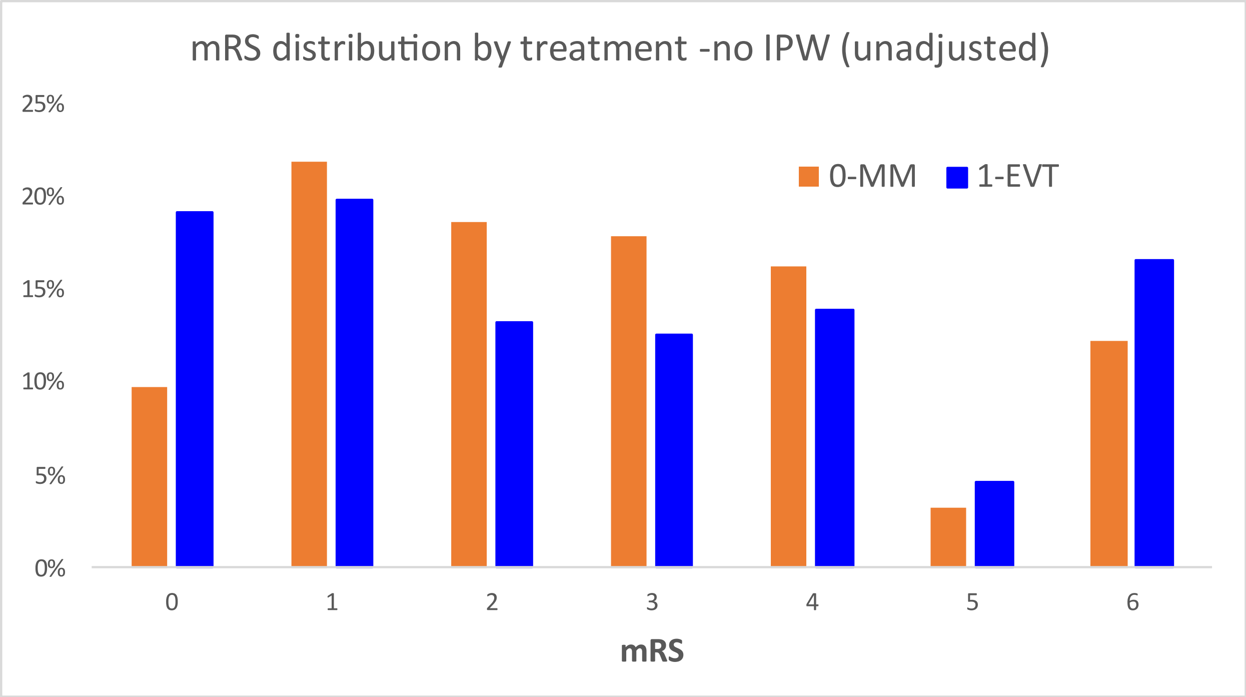

eFigure 1. Distribution of 90-Day mRS Scores by Treatment Group

IPTW: Inverse probability of treatment weighting. MM: Medical management. EVT: Endovascular therapy. mRS: modified Rankin scale.

© 2022 Saber H et al. *JAMA Network Open.*

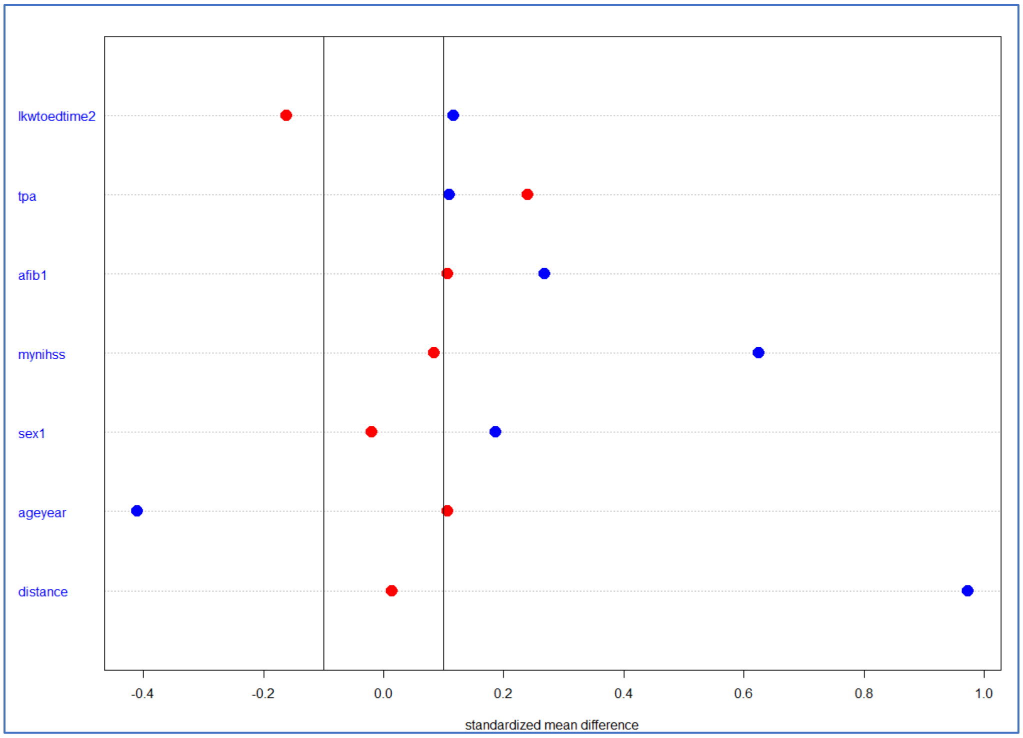

eFigure 2. Love Plot

blue: before IPTW, red: After IPTW.

IPTW: Inverse probability of treatment weighting.

© 2022 Saber H et al. *JAMA Network Open.*
